# Public concerns analysis and early warning of Mpox based on network data platforms—taking Baidu and WeChat as example

**DOI:** 10.3389/fpubh.2025.1523408

**Published:** 2025-03-05

**Authors:** Kai Yang, Shuangfeng Fan, Jiali Deng, Jinjie Xia, Xiaoyuan Hu, Linlin Yu, Bin Wang, Wei Yu

**Affiliations:** ^1^Chengdu Workstation for Emerging Infectious Disease Control and Prevention, Chinese Academy of Medical Sciences, Chengdu Center for Disease Control and Prevention, Chengdu, China; ^2^Department of Orthopaedics, The First Affiliated Hospital of Chengdu Medical College, Sichuan, China; ^3^Emergency Response Office, Xinjiang Uighur Autonomous Region Center for Disease Control and Prevention, Urumqi, China; ^4^Comprehensive Emergency Office, Center for Disease Control and Prevention of Qingbaijiang District, Chengdu, China

**Keywords:** Mpox, Baidu, WeChat, big data, public concern, early warning

## Abstract

With the outbreak of Mpox in non-endemic countries in May 2022, which has captured international attention. In response, this study leveraged the real-time, predictive, and wide coverage advantages of big data to reflect the public’s needs and interests regarding the Mpox epidemic, and explore its potential early warning role. We carried out a systematic data search weekly on two major network data platforms—Baidu Search Index (BDI) and WeChat Search Index (WCI) in China, and the index data overview, main concern information, hotspot regional distribution were analyzed. Besides, the correlation between the search index and the number of new cases of Mpox globally and within China were also investigated. Our results showed that both BDI and WCI mirrored the trends of the Mpox epidemic, with peaks in interest aligning with the release of relevant policies and events. The public’s interest evolved from basic knowledge of the disease to a focus on treatment and prevention, with attentiveness centrally placed in economically developed areas such as Guangdong, Beijing, and Shanghai. A positive correlation was observed between the Chinese epidemic and the BDI (*r* = 0.372, *p* = 0.047) and WCI (*r* = 0.398, *p* = 0.044), whereas non-correlation was noted globally. Notably, when the search time was delayed by 1 week, both BDI and WCI showed a positive correlation with the epidemic in China and globally. Overall, the integrated use of big data offers a platform for rapid understanding public concerns and early warning signs of emerging infectious diseases such as Mpox.

## 1 Introduction

Mpox, a zoonotic viral disease caused by the Mpox virus (MPXV). It shares similarities with smallpox in terms of symptoms, albeit with a milder presentation, which encompasses high fever, headache, lymphadenopathy, and pustules, with a mortality rate ranging between 1 and 10% (1, 2). The global health community has turned its focus toward Mpox following reports of cases in several non-endemic countries since May 2022 (3). As of August 31, 2024, the epidemic has spread across 123 countries, involving a total of 106,310 cases, marking it as the largest and most extensively spread nonendemic Mpox outbreak to date. Notably, this ongoing outbreak has unveiled novel epidemiological and clinical features (4–8), predominantly among men who have sex with men (MSM), though not exclusively (4, 9).

Traditionally, Mpox epidemic data have been primarily sourced from case management systems, which offers the benefit of data accuracy but lack immediacy (10). With the widespread accessibility of online resources and increasing global concern about Mpox, public awareness and interest in this disease have surged. Especially during the early stages of an unfamiliar epidemic, the public often exhibits anxiety and fear, and will first seek to understand what the disease is, how to protect against it, how to treat it and the extent of its harm (11, 12). Consequently, analyzing public attention has emerged as a novel approach to gauging the dynamics of this disease. Recently, many researchers have utilized big data platforms such as Google Trends and Twitter to identify patterns, trends, and sentiments related to infectious diseases, such as dengue fever, hand-foot-and-mouth disease, and H7N9, as well as to predict epidemic situations (13–15). These methods have also proven valuable in gauging public opinion, monitoring outbreaks, tracking vaccination efforts, and addressing disease-related concerns (16, 17). Additionally, this approach can compensate for the limitations of clinical data (18).

Baidu, a popular search engine among Chinese netizens, can largely reflect the current online search situation. Its subsidiary, the Baidu Search Index (BDI, http://index.baidu.com), a public data platform similar to Google Trends, parallels in aggregating keyword search data, and offering a window into public interest freely. Through the BDI, people can find search trends for selected terms, gain insights into changes in netizens’ needs, monitor media trends, and identify user characteristics. In recent years, instant messaging products such as WeChat have actively expanded the search, public account, and short video functionalities, providing a basis for increased attention given to certain events. Additionally, the embedded WeChat Search Index (WCI) mini-programs gather similar functions to the BDI. As common online platforms in China, the search data provided by the BDI and WCI can reflect the real needs and interests of the general public to some extent (19–22).

By leveraging real-time information, strong predictive capabilities, and wide coverage of big data, this study focused on the keyword “Mpox” in the BDI and WCI. This study retrospectively analyzed the hot topics of public concern, pinpointed the geographical areas of heightened interest, and explored the correlation between different stages of the Mpox epidemic and public attention. Furthermore, by innovatively integrating big data analysis with an evaluation of public interest in Mpox, this study also hopes to explore its potential early warning role.

## 2 Method

### 2.1 Keywords screening

To avoid discrepancies caused by the identification of different keywords, we utilized “Mpox” as the benchmark word and employed the original of keyword available on Chinaz,^1^ a free web platform for long-tail keyword searches, for keyword mining. Subsequently, we sequentially incorporated the top 20 keywords into both the BDI and WCI for inquiries. Considering the inclusion of keywords simultaneously by both platforms and the completion and validity of the time series of each keyword,“Mpox,” “MPXV,” and “MPXV vaccine” were ultimately selected for analysis.

### 2.2 Data sources

#### 2.2.1 Global Mpox cases

The global new cases of Mpox were sourced from the official website of the World Health Organization (WHO, https://www.who.int/), with a collection period from April, 2022, to August, 2024.

#### 2.2.2 Index-related data

The BDI-related data for Mpox were collected weekly from April 4, 2022, to September 30, 2024. To understand the impact of different events during the same period on people’s attention to Mpox, data with the keywords “COVID-19” and “novel coronavirus pneumonia” were collected during this period. Due to the limitations of data storage time on the WCI, the relevant data collection period for Mpox was from July 11, 2022, to September 30, 2024.

### 2.3 Index definition

#### 2.3.1 Baidu search index

It is a data-sharing platform based on the massive behavior data of Baidu netizens, which can dynamically reflect the search scale of a specific keyword on Baidu and its trend over a certain period, and reflect the public attention. The main modules include the search index, demand graph, related word popularity, and regional distribution.

#### 2.3.2 WeChat search index

The mobile terminal index is analyzed based on WeChat big data, including WeChat searches, public account articles, and publicly shared articles on Moments. This approach allows WeChat users to understand the popularity of a specific keyword within the WeChat ecosystem over a certain period of time. The main modules include search index, fluctuating data changes, and data sources.

### 2.4 Statistical analysis

Excel 2010(Microsoft Corp., WA, USA)was used to establish a database and organize the data, and togethered with GraphPad Prism of version 8 (GraphPad Software, Inc., La Jolla, CA, USA) for data analysis and related graphics construction. Spearman’s correlation coefficient was employed to evaluate the correlation between the search index and the number of new cases of Mpox worldwide and in China. The popularity of related terms and regional rankings were assigned a score of 1–10, with a higher score indicating greater attention. ArcGIS of version 10.5 (Environmental Systems Research Institute, Redlands, CA, USA) was utilized to map the regional distribution. A dotplot was generated with an online platform for data analysis and visualization.^2^ *p* < 0.05 indicated statistical significance.

## 3 Results

### 3.1 Analysis of BDI search trends

**Figure 1:**
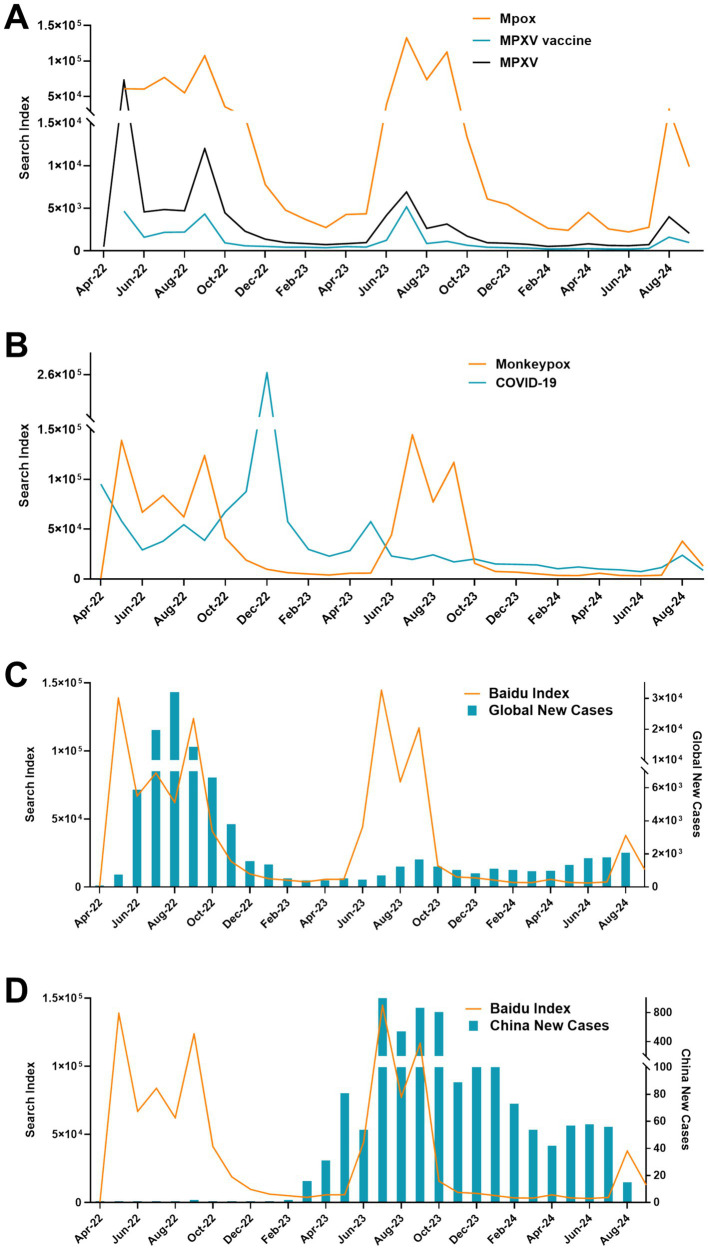
Overview of the Baidu search index. **(A)** The search index of each keyword associated with Mpox. **(B)** Comparative overview of the Mpox and COVID-19. **(C)** Overview of BDI and global new cases. **(D)** Overview of BDI and China new cases.

During this period, the BDI monitored a total of 1.06 million pieces of information. Upon conducting a comparative analysis of search trends for keywords associated with “Mpox,” it was discerned that each keyword manifests distinct peaks of attention at different times. Moreover, the overall trends associated with “Mpox,” “MPXV,” and “MPXV Vaccine” generally exhibited a consistent fluctuation trend (Figure 1A). Moreover, a juxtaposition of the aggregate search volumes for Mpox against those for COVID-19 yielded no discernible correlation (*t* = 0.274, *p* = 0.786). The peak values of Mpox were in May, and September of 2022, and July of 2023, while the peak of COVID-19 occurred in December 2022 (Figure 1B).

The analysis of BDI and new case profiles revealed that until June 2023, BDI and global Mpox new outbreaks almost showed the same trend (Figure 1C). Interestingly, the epidemic in China was in a disseminated situation during this period. From June to September of 2023, the BDI showed a consistent fluctuation trend with the new cases in China. However, since October 2023, BDI has tended to stabilize (Figure 1D).

### 3.2 Analysis of WCI search trends

**Figure 2:**
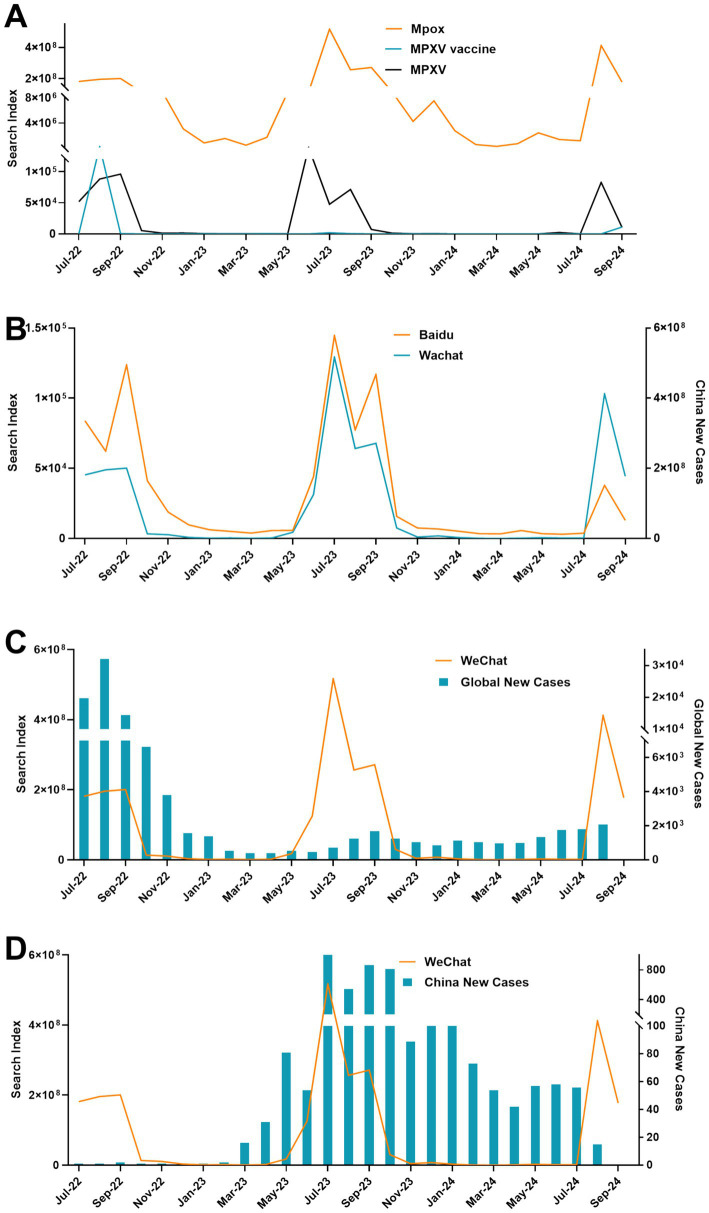
Overview of the WeChat search index. **(A)** The search index of each keyword associated with Mpox. **(B)** The overview comparison between the BDI and WCI. **(C)** Overview of WCI and global new cases. **(D)** Overview of WCI and China new cases.

During these years, the WCI has monitored a total of 2.44 billion pieces of information, and revealed that each keyword exhibited a basically consistent peak of attention, generally located around August and September 2022, as well as July 2023 and September 2024 (Figure 2A). When comparing the overall trends in BDI and WCI, netizens’ attention to Mpox were roughly the same (Figure 2B). In general, before April 2023, the WCI fluctuated along with the rise and fall of newly reported monkeypox cases globally. From May to September 2023, there was a sharp increase in search volume, during which time China experienced a outbreak of monkeypox. Afterward, the WCI showed a low and stable trend (Figures 2C,D).

### 3.3 Fluctuating data change analysis

**Figure 3:**
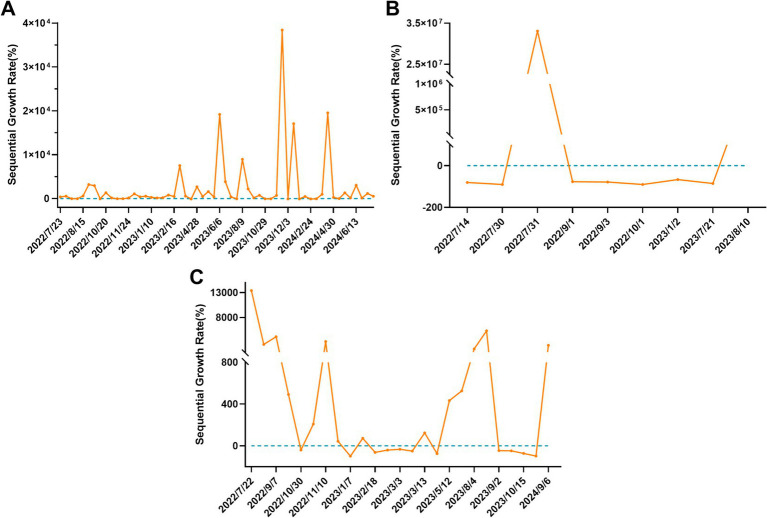
The fluctuating data change of WeChat search index. **(A)** Mpox. **(B)** MPXV. **(C)** MPXV vaccine.

The fluctuating data change reflects the diurnal ring ratio of public accounts, video accounts, searches, and web page data from the WeChat mobile terminal. The analysis showed that the significant variations in “Mpox” occurred in June and December of 2023, as well as in January and April of 2024 (Figure 3A). For “MPXV,” the duration changed greatly in July 2022 and August 2023. (Figure 3B). While for “MPXV Vaccine,” the variations were occurred in July, November of 2022 and August 2023, as well as in September 2024 (Figure 3C).

### 3.4 Demand graph analysis

**Figure 4:**
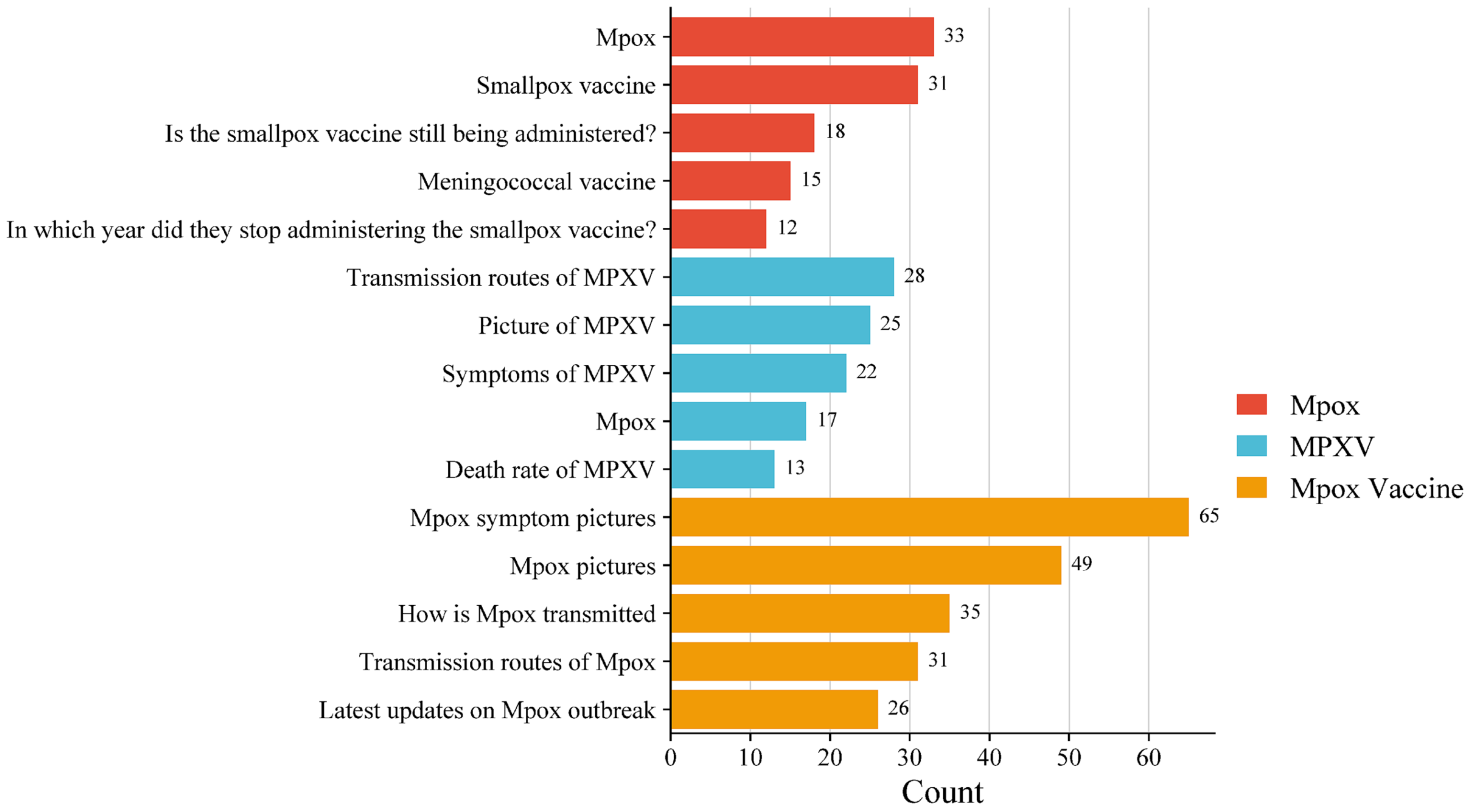
The demand graph associate to each keyword. The data was represented as the sum of the number of attention phrases associated with each keyword.

The demand graph mainly represents the relevant search term demands exhibited by users before and after searching for a particular term. The top 5 searched phrases with the most attention given to each keyword were listed (Figure 4). The analysis revealed 598 concerns related to “Mpox” among the netizens, mainly including “Mpox” (5.52%), “Smallpox vaccine” (5.19%), and “Is the small vaccine still being administered?” (0.30%). A total of 526 concerns for “MPXV” were found, mainly including “Transmission routes of MPXV” (5.32%), “Picture of MPXV” (4.75%), and “Mpox” (4.18%). In addition, 419 concerns for “MPXV Vaccine” were found, mainly including “Mpox symptom pictures” (15.51%), “Mpox pictures” (11.69%), and “How is Mpox transmitted” (8.35%).

### 3.5 Related word popularity analysis

**Figure 5:**
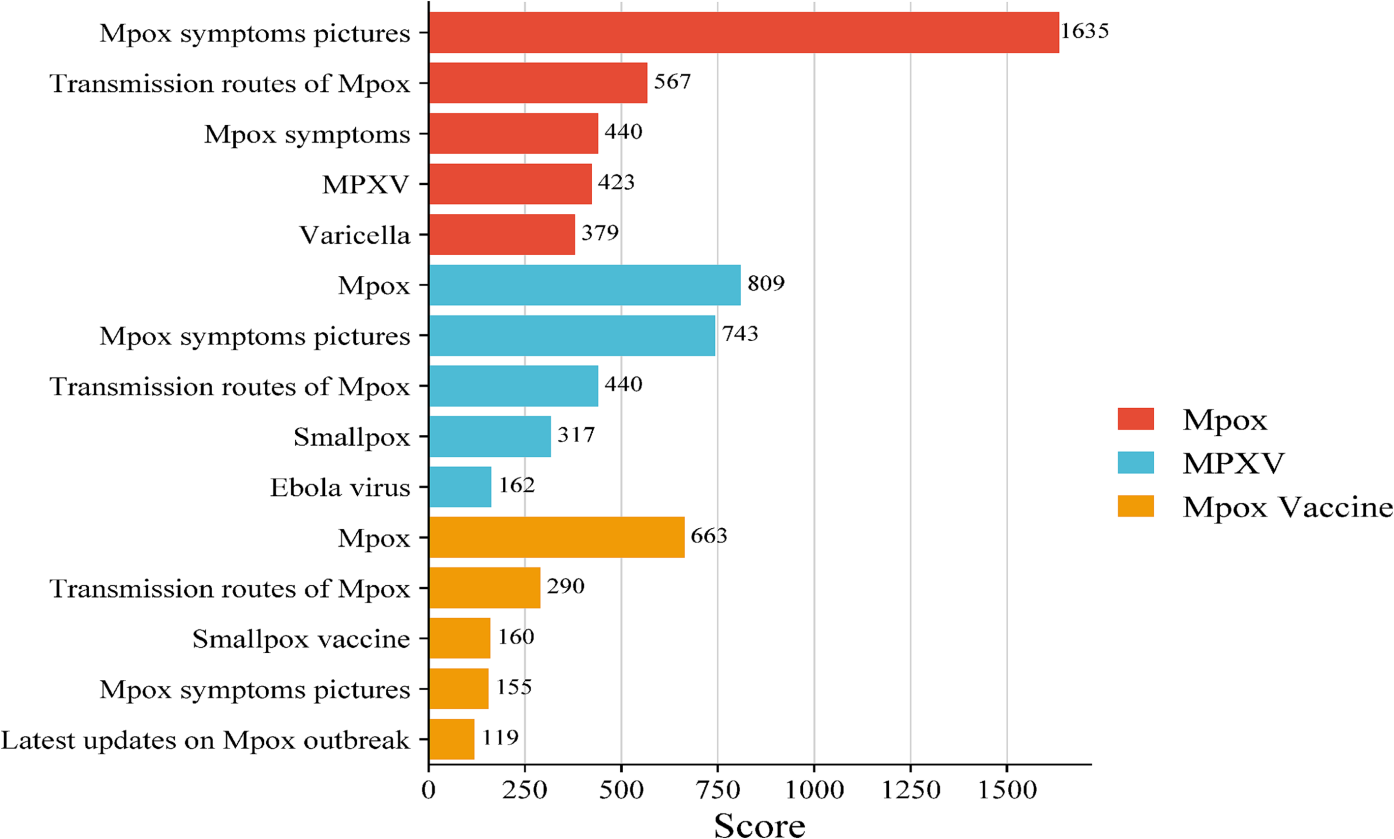
The hotspot words associated with each keyword. The hotspot word was assigned a score of 1–10 according to the ranking on a weekly basis. The higher the score was, the faster the attention given to the word increased during that week. The results were presented as the total weighted score of each hotspot word.

The related word popularity displays the related hotspot words in descending order of search popularity, reflecting the focus of public attention. By weighing and sorting the scores of each hotspot word, it was found that netizens paid attention to “Mpox” with concerns about “Mpox symptoms pictures,” “Transmission routes of Mpox,” and “Mpox symptoms” (Figure 5, red). For the concerns related to “MPXV,” the hotspot words were “Mpox,” “Mpox symptoms pictures,” and “Transmission routes of Mpox” (Figure 5, blue). While for “MPXV Vaccine,” the hotspot words were “Mpox,” “Transmission routes of Mpox,” and “Smallpox vaccine” (Figure 5, orange).

### 3.6 Geographic distribution

**Figure 6:**
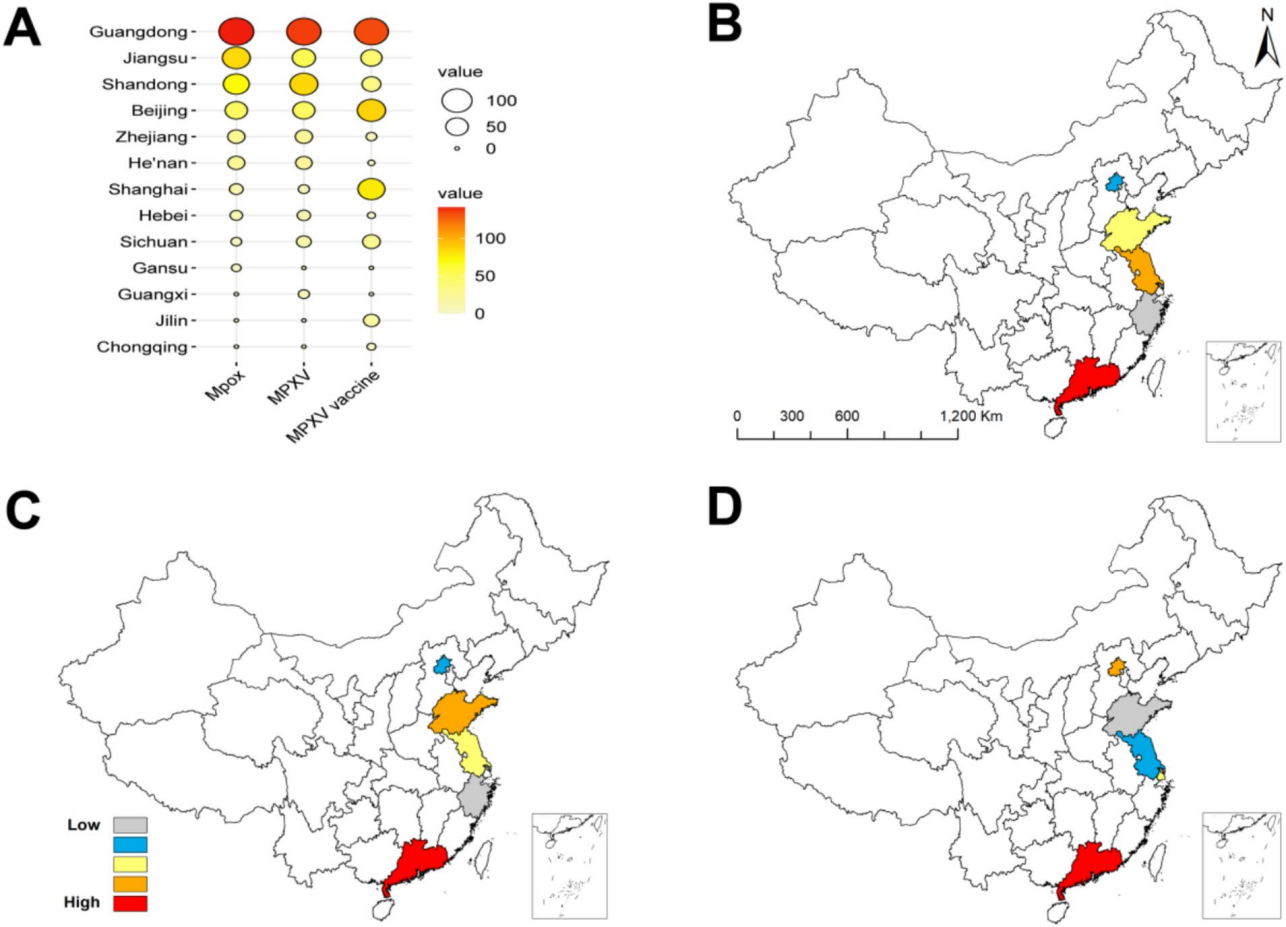
The geographical distribution of attention given to each keyword. **(A)** The distribution of the top ten provinces according to each keyword. **(B)** The distribution of the top five provinces according to “Mpox.” **(C)** The distribution of the top five provinces according to “MPXV.” **(D)** The distribution of the top five provinces according to “MPXV vaccine.”

The top 10 provinces with the most attention given to each keyword were collected monthly, and assigned a score ranging from 1 to 10 points based on the ranking. The overall weighted score of each keyword was shown in Figure 6A. In terms of “Mpox,” the provinces with high attention rankings were Guangdong, and Jiangsu (Figure 6B). For “MPXV,” the provinces were Guangdong, and Shandong (Figure 6C). While for “MPXV Vaccine,” the provinces were Guangdong, and Beijing (Figure 6D).

### 3.7 Correlation analysis

**Figure 7:**
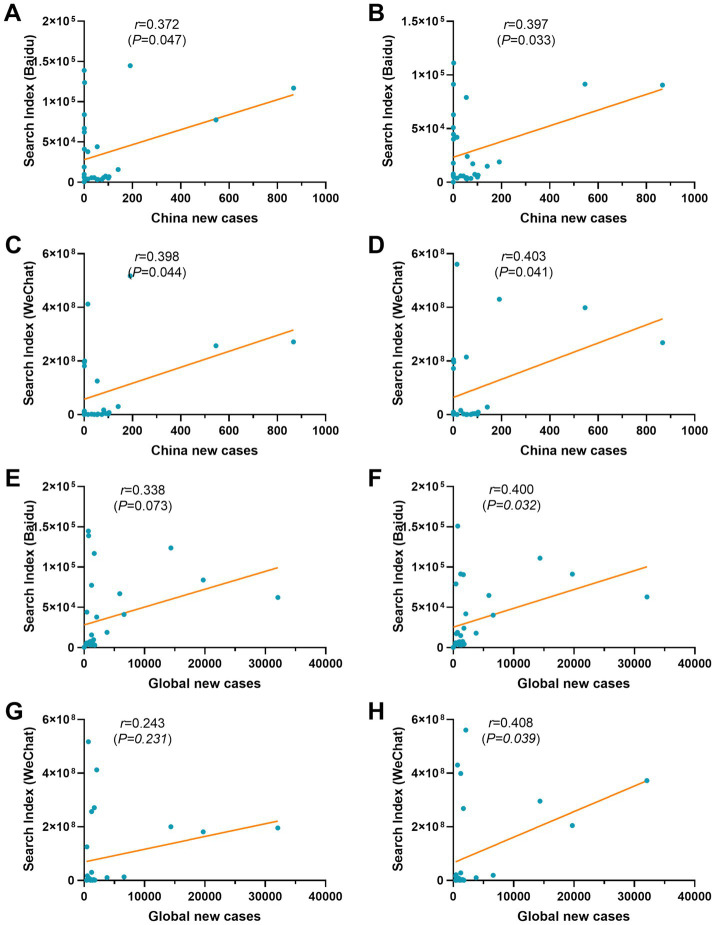
Correlation analysis between new cases and the search index. **(A)** New cases in China and BDI. **(B)** New cases in China and the lagged effect of BDI. **(C)** New cases in China and the WCI. **(D)** New cases in China and the delayed effect of WCI. **(E)** Global new cases and BDI. **(F)** Global new cases and the delayed effect of BDI. **(G)** Global new cases and WCI. **(H)** Global new cases and the delayed effect of WCI. The data were analyzed by Spearman’s correlation coefficient. *p* < 0.05 was considered to indicate statistical significance.

Correlation analysis between the search index and the number of new cases showed that both BDI (*r* = 0.372, *p* = 0.047) and WCI (*r* = 0.398, *p* = 0.044) exhibited a positive correlation between the number of new cases in China (Figures 7A,C). However, both BDI and WCI were not correlated with the number of new cases globally (Figures 7E,G). Considering the delay in issuing case announcements on the WHO website, a further analysis of the search index was conducted with a one-week lag. Interestingly, the results showed that both BDI and WCI present a positive correlation with the number of new cases in China and globally (Figures 7B,D,F,H).

## 4 Discussion

In May 2022, concurrent with the ongoing COVID-19 pandemic, the international community witnessed the widespread outbreak of Mpox (23, 24). This emerging infectious disease rapidly escalated into an event of international concern, raising the alert for global health security (9, 25). How to prevent Mpox has become the focus of public attention. To understand the trend of netizens’ attention to Mpox, this research advanced the monitoring timeframe to April 2022, and a total of 1.06 million pieces of information were monitored from BDI. The analysis revealed that the focus of netizens’ attention continuously changed with the status of the epidemic and the passage of time. In the early stages of the epidemic, people were in a state of confusion, with “what is Mpox” and “the transmission route of Mpox” becoming the content that people urgently seek to understand. Additionally, due to the intuitive understanding of Mpox, hot topics such as “Mpox picture” and “Mpox symptom picture” also increased during this stage. As time passed, the attention of netizens gradually shifted toward the prevention and treatment methodologies for Mpox.

The MPXV shares significant genetic and symptomatic similarities with smallpox virus. It is widely acknowledged that vaccination against smallpox can achieve a prevention rate of 85% for Mpox (26). In this study, we also found that while people paying attention to MPXV vaccines, there was also a notable uptick in searches related to smallpox, smallpox vaccines, vaccinia vaccine, and other related topics. However, many countries have stopped vaccinating against smallpox since the 1980s, resulting in an immunization gap for the younger generation. This may also be related to the fact that recent cases of Mpox have been concentrated in younger generations, which further highlights the urgency and importance of accelerating the development of MPXV vaccines. Currently, smallpox vaccines such as ACAM 2000 and Immune are approved for use against Mpox (27, 28), but the availability of specific MPXV vaccines remains limited.

Previous studies have shown that the trend evolution of online public opinion popularity was closely related to the progress of sudden public health emergencies (29, 30). Results of this study demonstrated that with the introduction of relevant policies and the release of events, the peak of the search index closely followed the significant progress of the epidemic. Notably, key moments such as the declaration of Mpox as a Public Health Emergency of International Concern (PHEIC) by WHO in July 2022, the classification of Mpox as a Class B infectious disease in China in September 2023, and the re-declaration as PHIEC, BDI and WCI both showed a peak along with these events. However, as time progresses since the occurrence of the events, the public becomes accustomed to it and their attention tends to stabilize. These research findings suggested that when major developments occur in international epidemics, not only should corresponding epidemic prevention and control measures be strengthened (31), but also relevant public opinion risks should be prevented. Furthermore, government and health departments should closely monitor the epidemic situation, and make full use of official websites or other channels to provide targeted education on Mpox basic knowledge, protective measures and other knowledge at different stages of the epidemic, so as to improve public protective measures and maintain public opinion order.

As of June 2024, the Chinese netizens has nearly reached 1.1 billion, with mobile phone users totaling 1.09 billion. With the increasing popularity of WeChat, it has gradually become the preferred chat tool for Chinese residents. In this study, the search index for Mpox on WeChat was far exceeded that on the BDI in terms of quantity. This may be attributed to WeChat’s high stickiness, multifunctional integration, and convenient search capabilities, which enable users to swiftly access desired information. Additionally, differences in data sources have contributed to the disparities between the BDI and WCI, but both showed consistent overall trends in attention for Mpox. These findings suggested that the use of multiple network platforms can more or less prevent information errors caused by single-platform analyses. This also provided new directions for the next stage of research by our research group. However, unfortunately, due to access limitations, a comparison of domestic online platforms with international platforms such as Twitter and Google remains unfeasible.

Related studies have shown that there were correlations between social media big data of the Google Trends, and Twitter, and epidemics, of H1N1 influenza, COVID-19, Ebola, Zika, dengue fever, and flu (32–35). Comparative analysis of new cases and search index reveals that BDI showed a consistent fluctuation trend with global new cases until June 2023, and thereafter with the Chinese epidemic (Figure 1). The same phenomenon also appeared in the change of WCI with the outbreak (Figure 2). In addition, we observed a positive correlation between both the BDI and WCI with China epidemics, yet an absence of correlation with epidemics globally. Interestingly, when the search time lag was extended by 1 week, the search index exhibited a positive correlation both in China and globally (Figure 7). On the one hand, this may be due to the relatively delayed publication of global Mpox epidemic data, coupled with the aggregation of public opinion information, potentially overshadowing the focus on domestic epidemics. On the other hand, this “low outbreak, high search volume” trend may also conceal the relevance of the results. Consequently, by delaying the search time, this situation was improved. In summary, leveraging information from online data platforms such as BDI and WCI can, to some extent, reflect epidemic trends and indirectly serve as a warning and alert for emerging infectious diseases like Mpox.

The distribution of populations reflects the overall attention given to a certain keyword in different regions during a certain period. This study revealed that regions with high overall attention given to Mpox were located in economically developed regions such as Guangdong, Beijing, and Shanghai, China. According to the 54nd Statistical Report of the China Internet Network Information Center (CNNIC; https://cnnic.cn/n4/2023/0828/c199-10830.html), the urban Internet penetration rate in China has reached 85.30%, and the convenience of obtaining information from urban residents and the higher demand for medical and public health have led to differences in regional attention. Additionally, economic variations across regions have prompted the migration of young people from western to eastern cities in China. Combined the dual factors of age and population size, this has also led to a higher level of attention in economically developed regions.

Furthermore, it is worth noting that Mpox has always been a neglected disease, predominantly affecting impoverished regions in Central and West Africa. It was not until 2003, when the first cases outside of Africa were identified in the United States, that Mpox garnered international attention (36, 37). Typically, poorer economic and sanitary conditions correlate with more frequent and widespread outbreaks (38, 39). Therefore, the allocation of health resources should not be based solely on the level of attention, as areas with high attention do not necessarily have higher priority. Proactively addressing these issues before they escalate is in the collective interest (8, 40, 41), meriting reflection. Similarly, China should optimize the allocation of resources for infectious diseases such as Mpox based on the varying needs of internet users.

This article has certain limitations. Firstly, the BDI is unable to analyze demographic characteristics beyond the month of data collection. Hence, age and gender demographic characteristics were not analyzed in this study. Secondly, a single data platform may only focus on data from specific regions or languages, and it may be difficult to integrate and analyze data from multiple platforms, leading to the phenomenon of information silos. To ensure the accuracy of the data, it is necessary to compare with other data platforms and consider the overall focus on different periods. Moreover, due to the sensitivity of the Mpox case data, the case data from various provinces has not been successfully obtained, thereby preventing further exploration of the correlation between public attention in each province and the number of cases. Lastly,we do not exclude the possibility that those who pay high attention to Mpox may include patients with Mpox or individuals at high risk, who are eager to learn about the basic knowledge and preventive and treatment measures of Mpox, and therefore choose to seek preliminary information through online platforms before seeking medical treatment. The search behavior of this group may potentially affect the quality and reliability of the data.

## 5 Conclusion

The innovative approach of combining big data platforms with evaluating public interest in Mpox provides valuable insights into public awareness, concepts, and concerns, as well as a reference for targeted regional propaganda. Moreover, the combination of organic big data and traditional monitoring methods can improve the monitoring and prediction capabilities of Mpox epidemics. We posit that leveraging big data can augment our response and communication strategies for emergent infectious diseases, thereby advancing public health outcomes and contributing to the “One Health” objective.

## Data Availability

The raw data supporting the conclusions of this article will be made available by the authors, without undue reservation.

## Footnotes

^1^ https://stool.chinaz.com/baidu/words.aspx

^2^ https://www.bioinformatics.com.cn

## References

[1] Ukwaja KN Hendris Izibewule J Ogunleye A Ederiane E Anebonam U Neni A . The 2017 human monkeypox outbreak in Nigeria—Report of outbreak experience and response in the Niger Delta University Teaching Hospital, Bayelsa State, Nigeria. PLoS One. (2019) 14:e0214229. doi: 10.1371/journal.pone.0214229, PMID: 30995249 PMC6469755

[2] Doshi RH Guagliardo SAJ Doty JB Babeaux AD Matheny A Burgado J . Epidemiologic and Ecologic Investigations of Monkeypox, Likouala Department, Republic of the Congo, 2017. Emerg Infect Dis. (2019) 25:281–9. doi: 10.3201/eid2502.181222, PMID: 30666937 PMC6346463

[3] Kozlov M. Monkeypox goes global: why scientists are on alert. Nature. (2022) 606:15–6. doi: 10.1038/d41586-022-01421-8, PMID: 35595996

[4] Patel A Bilinska J Tam JCH da Silva Fontoura D Mason CY Daunt A . Clinical features and novel presentations of human monkeypox in a central London centre during the 2022 outbreak: descriptive case series. BMJ. (2022):e072410. doi: 10.1136/bmj-2022-072410, PMID: 35902115 PMC9331915

[5] Tarín-Vicente EJ Alemany A Agud-Dios M Ubals M Suñer C Antón A . Clinical presentation and virological assessment of confirmed human monkeypox virus cases in Spain: a prospective observational cohort study. Lancet. (2022) 400:661–9. doi: 10.1016/S0140-6736(22)01436-2, PMID: 35952705 PMC9533900

[6] Català A Clavo-Escribano P Riera-Monroig J Martín-Ezquerra G Fernandez-Gonzalez P Revelles-Peñas L . Monkeypox outbreak in Spain: clinical and epidemiological findings in a prospective cross-sectional study of 185 cases. Br J Dermatol. (2022) 187:765–72. doi: 10.1111/bjd.21790, PMID: 35917191

[7] Thornhill JP Barkati S Walmsley S Rockstroh J Antinori A Harrison LB . Monkeypox virus infection in humans across 16 Countries — April–June 2022. N Engl J Med. (2022) 387:679–91. doi: 10.1056/NEJMoa2207323, PMID: 35866746

[8] Gessain A Nakoune E Yazdanpanah Y. Monkeypox. N Engl J Med. (2022) 387:1783–93. doi: 10.1056/NEJMra2208860, PMID: 36286263

[9] Kmiec D Kirchhoff F. Monkeypox: a new threat? Int J Mol Sci. (2022) 23:7866. doi: 10.3390/ijms23147866, PMID: 35887214 PMC9321130

[10] Milinovich GJ Williams GM Clements ACA Hu W. Internet-based surveillance systems for monitoring emerging infectious diseases. Lancet Infect Dis. (2014) 14:160–8. doi: 10.1016/S1473-3099(13)70244-5, PMID: 24290841 PMC7185571

[11] Chew C Eysenbach G. Pandemics in the age of Twitter: content analysis of tweets during the 2009 H1N1 outbreak. PLoS One. (2010) 5:e14118. doi: 10.1371/journal.pone.0014118, PMID: 21124761 PMC2993925

[12] Liu K Li L Jiang T Chen B Jiang Z Wang Z . Chinese public attention to the outbreak of Ebola in West Africa: evidence from the online big data platform. Int J Environ Res Public Health. (2016) 13:780. doi: 10.3390/ijerph13080780, PMID: 27527196 PMC4997466

[13] Guo P Wang L Zhang Y Luo G Zhang Y Deng C . Can internet search queries be used for dengue fever surveillance in China? Int J Infect Dis. (2017) 63:74–6. doi: 10.1016/j.ijid.2017.08.001, PMID: 28797591

[14] Xiao QY Liu HJ Feldman MW. Tracking and predicting hand, foot, and mouth disease (HFMD) epidemics in China by Baidu queries. Epidemiol Infect. (2017) 145:1699–707. doi: 10.1017/S0950268817000231, PMID: 28222831 PMC9203349

[15] Ruijing G Tan J Mo L Li Y Huang D. Using partial least squares regression to fit small data of H7N9 incidence based on the Baidu index. IEEE Access. (2020) 8:60392–400. doi: 10.1109/ACCESS.2020.2983799, PMID: 39573497

[16] Jiang B Zhu H Zhang J Yan C Shen R. Investor sentiment and stock returns during the COVID-19 pandemic. Front Psychol. (2021) 12:708537. doi: 10.3389/fpsyg.2021.708537, PMID: 34354650 PMC8329237

[17] Davidson MW Haim DA Radin JM. Using networks to combine “Big Data” and traditional surveillance to improve influenza predictions. Sci Rep. (2015) 5:8154. doi: 10.1038/srep08154, PMID: 25634021 PMC5389136

[18] Yang Y Li X Ma Q Fu Z Su K. Detecting epidemiological relevance of adenoid hypertrophy, rhinosinusitis, and allergic rhinitis through an Internet search. Eur Arch Otorrinolaringol. (2022) 279:1349–55. doi: 10.1007/s00405-021-06885-4, PMID: 34104981 PMC8187132

[19] Fan Z Yin W Zhang H Wang D Fan C Chen Z . COVID-19 information dissemination using the WeChat communication index: retrospective analysis study. J Med Internet Res. (2021) 23:e28563. doi: 10.2196/28563, PMID: 34129515 PMC8288647

[20] Zhao C Yang Y Wu S Wu W Xue H An K . Search trends and prediction of human brucellosis using Baidu index data from 2011 to 2018 in China. Sci Rep. (2020) 10:5896. doi: 10.1038/s41598-020-62517-7, PMID: 32246053 PMC7125199

[21] Qiu HJ Yuan LX Wu QW Zhou YQ Zheng R Huang XK . Using the internet search data to investigate symptom characteristics of COVID-19: A big data study. World J Otorhinolaryngol Head Neck Surg. (2020) 6:S40–8. doi: 10.1016/j.wjorl.2020.05.003, PMID: 32837757 PMC7236685

[22] Wang W Wang Y Zhang X Jia X Li Y Dang S. Using WeChat, a Chinese social Media App, for early detection of the COVID-19 Outbreak in December 2019: retrospective study. JMIR Mhealth Uhealth. (2020) 8:e19589. doi: 10.2196/19589, PMID: 32931439 PMC7572119

[23] Martín-Delgado MC Martín-Sánchez FJ Martínez-Sellés M Molero García JM Moreno Guillén S Rodríguez-Artalejo F . Monkeypox in humans: a new outbreak. Rev Esp Quimioter. (2022) 35:509–18. doi: 10.37201/req/059.2022, PMID: 35785957 PMC9728594

[24] Lahariya C Thakur A Dudeja N. Monkeypox disease outbreak (2022): epidemiology, challenges, and the way forward. Indian Pediatr. (2022) 59:636–42. doi: 10.1007/s13312-022-2578-2, PMID: 35762024 PMC9419123

[25] Gostin LO. Living in an age of pandemics—from COVID-19 to Monkeypox, Polio, and disease X. JAMA Health Forum. (2022) 3:e224062. doi: 10.1001/jamahealthforum.2022.4062, PMID: 36218949

[26] Fine PEM Jezek Z Grab B Dixon H. The transmission potential of Monkeypox virus in human populations. Int J Epidemiol. (1988) 17:643–50. doi: 10.1093/ije/17.3.643, PMID: 2850277

[27] Petersen BW Kabamba J McCollum AM Lushima RS Wemakoy EO Muyembe Tamfum JJ . Vaccinating against monkeypox in the Democratic Republic of the Congo. Antivir Res. (2019) 162:171–7. doi: 10.1016/j.antiviral.2018.11.004, PMID: 30445121 PMC6438175

[28] See KC. Vaccination for Monkeypox virus infection in humans: a review of key considerations. Vaccine. (2022) 10:1342. doi: 10.3390/vaccines10081342, PMID: 36016230 PMC9413102

[29] Zhang S Chu-ke C Kim H Jing C. Public view of public health emergencies based on artificial intelligence data. J Environ Public Health. (2022) 2022:5162840. doi: 10.1155/2022/5162840, PMID: 36034623 PMC9410812

[30] Liu J Qi J. Online public rumor engagement model and intervention strategy in major public health emergencies: from the perspective of social psychological stress. Int J Environ Res Public Health. (2022) 19:1988. doi: 10.3390/ijerph19041988, PMID: 35206175 PMC8871882

[31] Lahariya C. Preventive medicine: initium salutis renaissance. Prev Med Res Rev. (2024) 1:1–3. doi: 10.4103/PMRR.PMRR_1_23

[32] Ho HT Carvajal TM Bautista JR Capistrano JDR Viacrusis KM Hernandez LFT . Using Google trends to examine the spatio-temporal incidence and behavioral patterns of dengue disease: a case study in Metropolitan Manila, Philippines. Trop Med Infect Dis. (2018) 3:118. doi: 10.3390/tropicalmed3040118, PMID: 30423898 PMC6306840

[33] Signorini A Segre AM Polgreen PM. The use of Twitter to track levels of disease activity and public concern in the U.S. during the influenza A H1N1 pandemic. PLoS One. (2011) 6:e19467. doi: 10.1371/journal.pone.0019467, PMID: 21573238 PMC3087759

[34] Milinovich GJ Magalhães RJS Hu W. Role of big data in the early detection of Ebola and other emerging infectious diseases. Lancet Glob Health. (2015) 3:e20–1. doi: 10.1016/S2214-109X(14)70356-0, PMID: 25539964

[35] Paul R Teng Y Bi D Xie G Jin Y Huang Y . Dynamic forecasting of Zika epidemics using Google trends. PLoS One. (2017) 12:e0165085. doi: 10.1371/journal.pone.0165085, PMID: 28060809 PMC5217860

[36] Sale TA Melski JW Stratman EJ. Monkeypox: An epidemiologic and clinical comparison of African and US disease. J Am Acad Dermatol. (2006) 55:478–81. doi: 10.1016/j.jaad.2006.05.061, PMID: 16908354 PMC9629018

[37] Reed KD Melski JW Graham MB Regnery RL Sotir MJ Wegner MV . The detection of monkeypox in humans in the Western Hemisphere. N Engl J Med. (2004) 350:342–50. doi: 10.1056/NEJMoa032299, PMID: 14736926

[38] Gromowski G Bunge EM Hoet B Chen L Lienert F Weidenthaler H . The changing epidemiology of human monkeypox—A potential threat? A systematic review. PLoS Negl Trop Dis. (2022) 16:e0010141. doi: 10.1371/journal.pntd.0010141, PMID: 35148313 PMC8870502

[39] McNabb SJN Chungong S Ryan M Wuhib T Nsubuga P Alemu W . Conceptual framework of public health surveillance and action and its application in health sector reform. BMC Public Health. (2022) 2:1–9. doi: 10.1186/1471-2458-2-2 PMID: 11846889 PMC65598

[40] Nakoune E Olliaro P. Waking up to Monkeypox. BMJ. (2022) 377:O1321. doi: 10.1136/bmj.o1321 35613732

[41] Jayasinghe M Caldera D Prathiraja O Kayani AMA Siddiqui OS Coffie-Pierre JA . Waking up to Monkeypox in the midst of COVID-19. Cureus. (2022) 14:e30920. doi: 10.7759/cureus.30920, PMID: 36465725 PMC9710728

